# 

**Published:** 1889-11-30

**Authors:** 


SATURDAY, NOVEMBER 30th, 1889.

**Miss Cobbe's Doctors** - 129

**Words of Consolation**—"Light" - 130

**The Doctor's Pulpit.**—Physiological Candles - 131

**A Few Old Simples** - 132

**Some Unpleasant Truths about Food and Luxuries** 133

**Cremation and Urn-Burial** - 134

**Everybody's Page** - 135

**Notes and News** - 136

**Annotations.**—The Privilege of the Rich—A Debtor to his Profession—Children's Underclothing - 138

**Leprosy and Leper Houses** - 143

**The Practitioner's Outlook and Retrospect:**
MEDICINE—Leprosy - 139
The Hyderabad Commission on the Action of Chloroform 140
SURGERY—Wounds of the Brain - 141
THERAPEUTICS—Treatment of Dysentery—Use of Tobacco 141
NEW DRUGS, APPLIANCES, AND THINGS MEDICAL—Digby's Concentrated Soup Biscuit—Lister's New Antiseptic Dressings - 142
THE PRACTITIONER'S BOOKSHELF—A Textbook of General Therapeutics - 142
**Student's Column—Scraps & Gleanings** - 144

EXTRA SUPPLEMENT—THE NURSING MIRROR.

**En Passant.**—Short Items—Every Woman a Nurse—Nurses and Health—Our Correspondents—Manchester District Work—On the Continent - xxxvii
**The National Pension Fund for Nurses** - xxxviii
**Ward Decoration** - xxxviii
**Lectures—Notices** - xxxix
**Everybody's Opinion.**—Nursing at Netley—The Pension Fund—Ladies as District Nurses—The Beginning of Mildmay—Voting by Proxy at Brighton—The Scandal at Hope Infirmary—A Registry for Nurses - xxxix
**Presentations—Notes and Queries** - xl
**Appointments—Vacancies—Amusements** - xl

